# Transcriptomics Reveals the Mechanism of *Rosa roxburghii* Tratt Ellagitannin in Improving Hepatic Lipid Metabolism Disorder in db/db Mice

**DOI:** 10.3390/nu15194187

**Published:** 2023-09-28

**Authors:** Yunyun Tan, Shuming Tan, Tingyuan Ren, Lu Yu, Pei Li, Guofang Xie, Chao Chen, Meng Yuan, Qing Xu, Zhen Chen

**Affiliations:** 1 School of Liquor and Food Engineering, Guizhou University, Guiyang 550025, China; 2 The Key Laboratory of Plant Resources Conservation and Germplasm Innovation in Mountainous Region (Ministry of Education), Institute of Agro-Bioengineering and College of Life Sciences, Guizhou University, Guiyang 550025, China; 3 Qiandongnan Engineering and Technology Research Center for Comprehensive Utilization of National Medicine, Kaili University, Kaili 556018, China

**Keywords:** *Rosa roxburghii* Tratt, ellagitannin, transcriptomics, type 2 diabetes, hepatic lipid metabolism

## Abstract

A complex metabolic disorder, type 2 diabetes, was investigated to explore the impact of ellagitannin, derived from *Rosa roxburghii* Tratt (RTT), on liver lipid metabolism disorders in db/db mice. The findings demonstrated that both RTT ellagitannin (C1) and RTT ellagic acid (C4) considerably decelerated body mass gain in db/db mice, significantly decreased fasting blood glucose (FBG) levels, and mitigated the aggregation of hepatic lipid droplets. At LDL-C levels, C1 performed substantially better than the C4 group, exhibiting no significant difference compared to the P (positive control) group. An RNA-seq analysis further disclosed that 1245 differentially expressed genes were identified in the livers of experimental mice following the C1 intervention. The GO and KEGG enrichment analysis revealed that, under ellagitannin intervention, numerous differentially expressed genes were significantly enriched in fatty acid metabolic processes, the PPAR signaling pathway, fatty acid degradation, fatty acid synthesis, and other lipid metabolism-related pathways. The qRT-PCR and Western blot analysis results indicated that RTT ellagitannin notably upregulated the gene and protein expression levels of peroxisome proliferator-activated receptor alpha (PPARα) and peroxisome proliferator-activated receptor gamma (PPARγ). In contrast, it downregulated the gene and protein expression levels of sterol regulatory element-binding protein (SREBP), recombinant fatty acid synthase (FASN), and acetyl-CoA carboxylase (ACC). Therefore, RTT ellagitannin can activate the PPAR signaling pathway, inhibit fatty acid uptake and de novo synthesis, and ameliorate hepatic lipid metabolism disorder in db/db mice, thus potentially aiding in maintaining lipid homeostasis in type 2 diabetes.

## 1. Introduction

Numerous metabolic disorders, such as inadequate insulin secretion, elevated blood glucose levels, and irregular lipid profiles, are linked to type 2 diabetes [1]. As dietary levels change (such as heavy meat and fish consumption and overeating), the prevalence of diabetes gradually increases and is considered a chronic disease that poses a serious threat to the health of a large part of the population. As reported by the International Diabetes Federation (IDF), the global population of individuals with diabetes was estimated to reach 463 million in 2019 and is projected to increase to 700 million by 2045 [2]. Presently, the main approach to treating type 2 diabetes involves using oral hypoglycemic medications (such as metformin, acarbose, and rosiglitazone) to reduce blood sugar levels. However, several side effects have been associated with their long-term use, including headaches, nausea, diarrhea, hepatotoxicity, and more [3]. Consequently, there is an urgent need to identify natural active compounds from plants that can effectively treat diabetes.

*Rosa roxburghii* Tratt (RTT) belongs to the Rosaceae family as a perennial rose genus that is predominantly found in Southwest China [4,5]. RTT, a distinctive fruit native to Guizhou, is abundant in vitamin C, polyphenols, flavonoids, organic acids, and other bioactive constituents [6]. Xiang et al. [7] discovered that polyphenols, such as ellagic acid and ellagitannins, present in RTT can inhibit DPPH free radicals and exhibit varying degrees of ABTS free radical scavenging capacity, with anti-aging, hypoglycemic, anticancer, and anti-radiation effects [8,9,10]. Ellagic acid primarily exists as a condensed form of ellagitannin, having a smaller molecular weight than ellagitannin and functioning as a dimeric derivative of gallic acid [11]. Research has demonstrated that ellagic acid not only ameliorates hyperglycemia in STZ-induced diabetes, lowers blood glucose levels, and attenuates neuroinflammation [12] but also enhances the expression of glucose transporter protein (GLUT4) and PPARγ in skeletal muscle, safeguards pancreatic β-cells against reactive oxygen species, and improves insulin resistance [13]. Ahad et al. [14] identified the antioxidant and anti-inflammatory properties of ellagic acid, which significantly improved diabetes-induced nephropathy, inhibited nuclear factor-k-gene binding (NF-Kb) activity, stimulated anti-inflammatory factor synthesis, and reduced oxidative stress in kidney tissues. Conversely, Bodis et al. [15] found that ellagitannins may interact with proteins, alter enzyme conformations, and decrease α-glucosidase activity. Animal studies revealed that ellagitannins can reduce blood glucose levels in STZ-induced diabetic rats. According to a previous study, RTT ellagitannin can diminish glucokinase and glucose-6-phosphatase activities in type 2 diabetic mice, exerting a significant hypoglycemic effect [16]. However, the precise regulation and mechanism of RTT ellagitannin on lipid metabolism disorders in type 2 diabetes remain uncertain.

Lipid metabolism primarily encompasses *de novo* lipid synthesis (DNL), cholesterol metabolism, and lipid uptake and transport [17]. The structure of a lipid is determined by its fatty acid composition, which is closely related to insulin resistance in diabetes and mainly arises from dietary intake and endogenous DNL [18,19]. DNL is an essential process for converting carbohydrates into lipids, characterized by the release of acetyl-CoA from glucose through glycolysis into the citrate cycle and the subsequent release of acetyl-CoA. Acetyl-CoA is further catalyzed and converted to malonyl coenzyme A by the rate-limiting enzyme ACC. Malonyl coenzyme A serves as a substrate for FASN, an enzyme responsible for ab initio synthesis of palmitoleate; palmitoleate then undergoes a series of catalytic desaturation reactions to generate complex fatty acids, such as stearic acid, palmitoleic acid, and oleic acid [20,21,22]. A crucial transcription factor for DNL and cholesterol synthesis is sterol regulatory element-binding protein (SREBP) [15,23]. The terminal structural domains of SREBPs are hydrolyzed and enter the nucleus to regulate their involvement in de novo fatty acid synthesis by binding to various sites within the promoter regions of ACC and FASN genes [24,25].

Thus, in this research, we employed db/db mice to investigate the regulatory mechanism of RTT ellagitannin on lipid metabolism, using high-throughput sequencing (RNA-seq), aiming to provide a scientific foundation for its potential to ameliorate metabolic disorders in type 2 diabetes.

## 2. Materials and Methods

### 2.1. Materials

RTT ellagitannin (C1, HPLC ≥ 82%) and RTT ellagic acid (C4, HPLC ≥ 62%) were acquired from our group, utilizing the extraction method in accordance with the technique described by Chen et al. [15]. Fresh RTT was vacuum-dried, ground into a powder, extracted with 70% ethanol and 0.25% cellulase, purified using macroporous resin, and further extracted with ethyl acetate. Subsequently, TBE-300 B high-speed countercurrent chromatography was employed to separate the extracts, yielding ellagitannin and ellagic acid. C57/BKS-db/db and C57/BKS-db/m mice were obtained from Nanjing Gem Pharmaceutical Technology Co., Ltd. of China (Nanjing, China) (Animal Production License No. SYXK(Qian)2021–0005).

### 2.2. Animal Feeding Methods

Four-week-old C57/BKS-db/db and C57/BKS-db/m mice were housed in the Guizhou University animal facility, with a well-functioning ventilation system, maintaining 50–65% humidity and a room temperature of 22 ± 2 °C. The Animal Experimentation Ethics Committee of Guizhou University approved all experiments (No. EAE-GUZ-2020-P010). After mice acclimated for one week, db/m mice were used as the blank group (N), and db/db mice were divided into four groups (6 mice each): a model (M group), positive control (metformin hydrochloride gavage dose 50 mg/kg, P group), RTT ellagitannin (gavage dose 50 mg/kg [12], C1 group), and RTT ellagic acid (gavage dose 50 mg/kg, C4 group). Throughout the experimental period, mice were provided with standard chow and water ad libitum. Their growth was monitored, and their body weight was recorded weekly. Fasting blood glucose (FBG) levels were measured at 0, 4, and 8 weeks. After 8 weeks of continuous gavage intervention, each group fasted without water for 12 h. Mice were then euthanized following orbital blood collection and dissection. Subsequently, liver tissue was divided into two parts: one part was fixed with paraformaldehyde fixative for pathological observation, while the other part was preserved at −80 °C in liquid nitrogen.

### 2.3. Effect of RTT Ellagitannin on Liver Lipid Levels in Mice

Total cholesterol (TC), total triglyceride (TG), low-density lipoprotein cholesterol (LDL-C), and high-density lipoprotein cholesterol (HDL-C) were assessed using kits obtained from Nanjing Jiancheng Institute of Biological Engineering (Nanjing, China).

### 2.4. Pathological Analysis of Oil Red O

Liver sections were removed from the paraformaldehyde fixative, rinsed with water, dried, and stained with Oil Red O for 10 min. Subsequently, the liver sections were immersed in 60% isopropyl alcohol for differentiation twice (3 and 5 s each), rinsed in pure water twice for 10 s each, and stained with hematoxylin for 5 min. The sections were then rinsed in pure water thrice (5, 10, and 30 s each), differentiated in 60% alcohol for 5 s, washed in pure water twice for 10 s each, immersed in a blue solution for 1 s, and rinsed in distilled water twice (not exceeding 15 s each). Lastly, the liver sections were mounted with glycerol gelatin.

### 2.5. Transcriptome High-Throughput Sequencing

The samples were sequenced by Chengdu Nomi Metabolic Biotechnology Co., Ltd. (Chengdu, China). In the total RNA, Oligo(dT) magnetic beads were employed to enrich polyA-structured mRNA, and ion interruption fragmented RNA to approximately 300 bp. Conventional primers and reverse transcriptase were utilized to synthesize the first strand of cDNA, using RNA as a template, which was then used as a template for synthesizing the second strand. A Bioanalyzer Agilent 2100 (Waldbronn, Germany) ensured quality control of library fragment enrichment through PCR amplification. RNA was extracted and purified, and libraries were constructed, followed by double-end library sequencing based on Illumina novaseq6000 sequencing platform for bipartite library sequencing, with a sequencing data volume of 6 g and a read length of 250 bp.

### 2.6. qRT-PCR Analysis

Total RNAs of liver tissue were extracted using the TRIzol reagent kit (TaKaRa, Dalian, China). RNA was reverse transcribed using a cDNA kit (TaKaRa, Dalian, China) according to the manufacturer’s instructions. qRT-PCR was conducted using a 20 µL reaction sample with SYBR GREEN II (TaKaRa, Dalian, China) and the iCycleriQ multicolor real-time PCR detection system (Bio-Rad, Hercules, CA, USA). Gene expression was normalized using β-actin as an internal control. The primer sequences for peroxisome proliferator-activated receptor alpha (PPARα), peroxisome proliferator-activated receptor gamma (PPARγ), sterol regulatory element binding protein (SREBP), recombinant fatty acid synthase (FASN), and acetyl-CoA carboxylase (ACC) for qRT-PCR are shown in Table 1. The target genes were normalized by 2^−ΔΔCt^ results and expressed as relative gene expression levels [26,27].

### 2.7. Western Blot Analysis

Liver tissue blocks were washed with precooled phosphate buffer (PBS) to remove blood stains until the solution was clear, and then they were cut into small pieces and homogenized. The radioimmunoprecipitation assay (RIPA) lysis solution (ready-to-use) was used for homogenization in an ice bath, and the supernatant was collected by centrifugation at 12,000× *g* for 10 min to obtain the total protein. The protein concentration was measured using the bicinchoninic acid (BCA) method, and then *N*,*N*,*N*,*N*-tetramethylethylenediamine (TEMED) was added to mix the gel. After 30 min of liquid sealing with ddH_2_O, 10% ammonium persulfate (AP) and TEMED were both added, and the mixture was stirred for 30 min. Sodium dodecyl sulfate–polyacrylamide gel electrophoresis (SDS-PAGE) was stopped upon the appearance of bromophenol blue, and the membrane was transferred to a PVDF membrane for detection.

### 2.8. Data Analysis

All experiments were repeated more than three times, and the results were expressed as “mean ± standard deviation (SD)”. Statistical analysis was performed using SPSS 25.0 software to process and organize the data. A one-way ANOVA was used, followed by Dunnett’s post hoc test for normally distributed data. Subsequently, multiple data means were rank ordered for comparison and indicated using different lowercase letters. A probability of *p* < 0.05 indicated that the differences were significant and statistically significant.

## 3. Results

### 3.1. Effect of RTT Ellagitannin on Body Weight and FBG in Mice 

The characteristic feature of type 2 diabetes is an initial weight gain that is followed by weight loss and thinning in mice. Throughout the experiment, the mice exhibited healthy growth with no mortality. Table 2 demonstrates that the body weights of mice in M, P, C1, and C4 groups were notably higher than those in the N group. After an 8-week intervention, the body weights of P, C1, and C4 groups decreased by 7.33%, 8.77%, and 5.18%, respectively, compared to the M group. Concurrently, Table 3 indicates that the FBG levels were higher in the M, P, C1, and C4 groups than in the N group during the feeding period. In contrast, compared to the M group, FBG in both C1 and C4 groups showed a significant decrease in the fourth week, while the P group exhibited an upward trend, although not statistically significant. After 8 weeks, the FBG of P, C1, and C4 groups significantly decreased by 31.42%, 52.27%, and 46.12%, respectively, in comparison to the M group. These results suggest that RTT ellagitannin can effectively reduce body weight and FBG in db/db mice.

### 3.2. Effect of RTT Ellagitannin on Liver Lipid Contents in Mice

**Figure 1:**
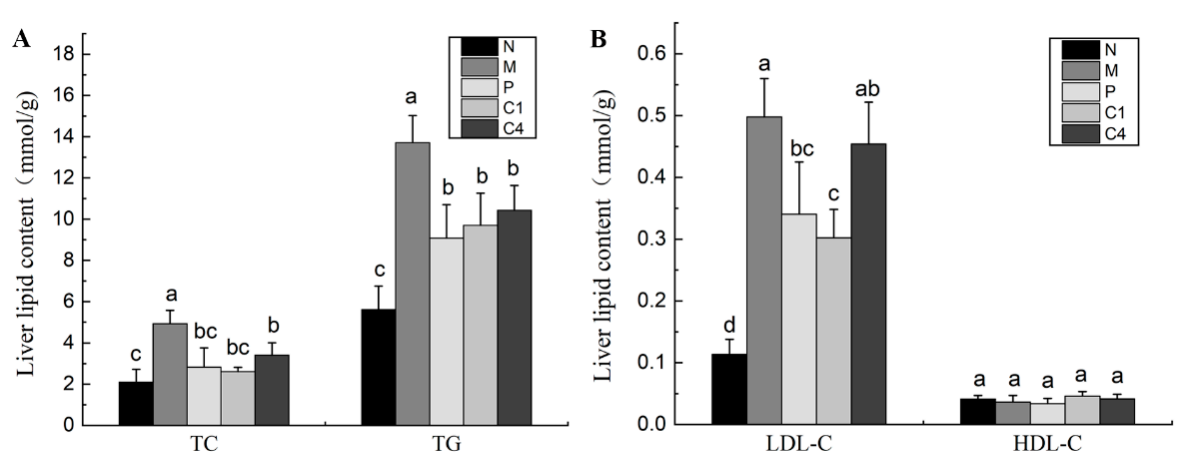
Effect of RTT ellagitannin on liver lipid levels in mice. (**A**) TC and TG contents. (**B**) LDL-C and HDL-C contents. All data are presented as mean ± SD (*n* = 6). Different lowercase letters indicate significant differences in TC, TG, LDL-C, and HDL-C contents at *p* < 0.05.

Figure 1A illustrates that, compared to the N group, the TC content in group M increased significantly by 135.99%; and compared to the M group, the TC content in groups P, C1, and C4 decreased by 42.70%, 47.09%, and 30.95%, respectively, indicating that C1 and C4 could significantly reduce the TC content, and improvement effect of C1 was better than that of group P, and the improvement effect of group P was better than that of C4, but there were no significant differences among (*p* > 0.05) the P, C1, and C4 groups. Compared to the N group, the TG content in group M increased significantly by 144.02%; the liver TG contents of the experimental mice (P, C1, and C4) all decreased significantly (*p* < 0.05) compared with group M, by 33.82%, 39.29, and 8.78%, respectively; however, there was no significant (*p* > 0.05) difference between groups P, C1, and C4, but the TG levels of C1 and P were close to the level of group N. Figure 1B demonstrates a 155.49% increase in the LDL-C content in the M group compared to the N group. P and C1 significantly reduced LDL-C levels by 31.60% and 36.57%, respectively, compared to the M group. C4 was not significant (*p* < 0.05) in mitigating LDL-C, but it had a decreasing trend. However, no significant differences were observed in the HDL-C contents among the groups. Thus, RTT ellagitannin was more effective in reducing the levels of TC, TG, and LDL-C, thereby ameliorating lipid metabolism disorders in type 2 diabetes mice. In the LDL-C level, C1 improved significantly better than C4.

### 3.3. Oil Red O Staining

**Figure 2:**
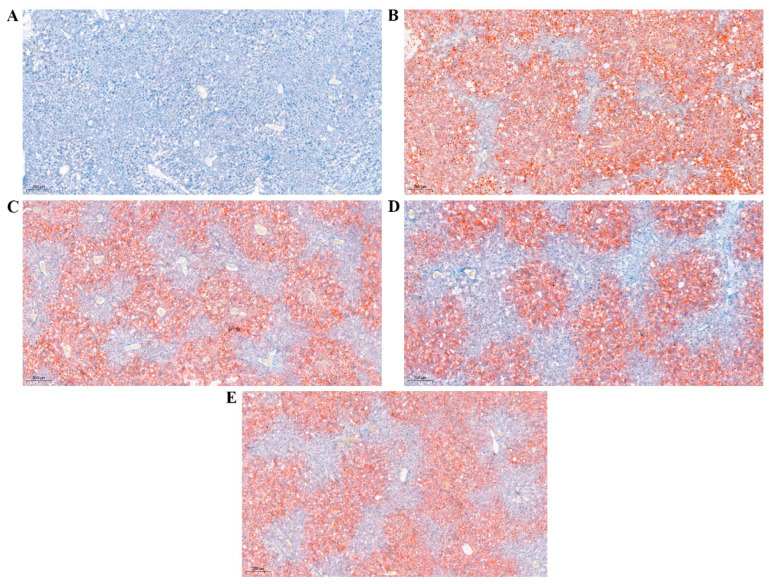
Representative Oil Red O-stained (magnification, ×200) sections of the livers in mice. (**A**) N group, (**B**) M group, (**C**) P group, (**D**) C1 group, and (**E**) C4 group.

Figure 2 demonstrates that, compared to the N group, the M group exhibited severe intracytoplasmic lesions in hepatocytes, with extensive liver tissue occupied by large circular lipid droplets. In contrast, the experimental intervention group displayed reduced lipid droplet deposition compared to the M group, with more effective alleviation of lipid accumulation in the liver observed in the P and C1 treated groups, and less improvement in the C4-treated group. Moreover, RTT ellagitannin effectively ameliorated lipid deposition symptoms in liver tissue and decreased the risk of liver steatosis.

### 3.4. Sequencing QC Filtering Analysis

The samples were up-sequenced to generate raw data, and the bases were recognized in the raw data of each sample; as shown in Table 4, the Q20 value of each sample was greater than 97%, and the value of Q30 was greater than 93%. This indicates that the samples are of high quality and that the data are credible and can be used for the next step of the analysis.

### 3.5. High-Throughput Sequencing Analysis

**Figure 3:**
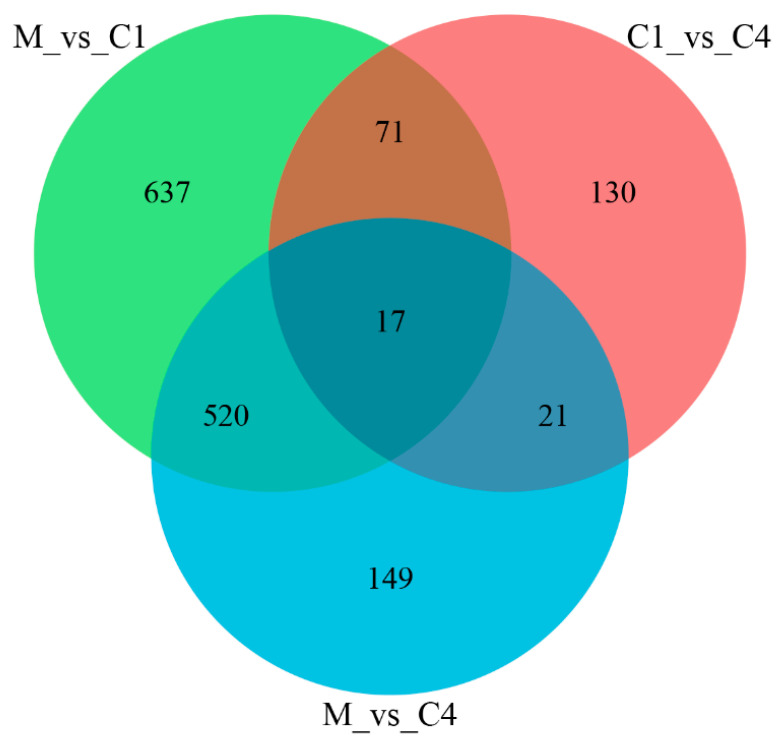
The Venn diagram of DEGs among M, C1, and C4 groups.

In M vs. C1, a total of 1245 differentially expressed genes (DEGs; log_2_FoldChange > 1, *p* < 0.05) were observed, with 695 DEGs significantly upregulated and 550 DEGs significantly downregulated. In M vs. C4, 707 DEGs were identified, including 366 significantly upregulated DEGs and 341 significantly downregulated DEGs (Figure 3).

### 3.6. GO Term and KEGG Pathway Analysis of DEGs

The GO enrichment analysis classifies DEGs with the same or similar functions into one category, primarily divided into the biological process (BP), cellular component (CC), and molecular function (MF). Based on the highest GO score, an enrichment analysis was conducted, calculating the DEGs associated with each GO term, using the gene list for each term. The top 30 GO term entries with the smallest *p*-value in the GO classification were selected. The most significantly enriched terms in the GO classification were chosen for enrichment display.

**Figure 4:**
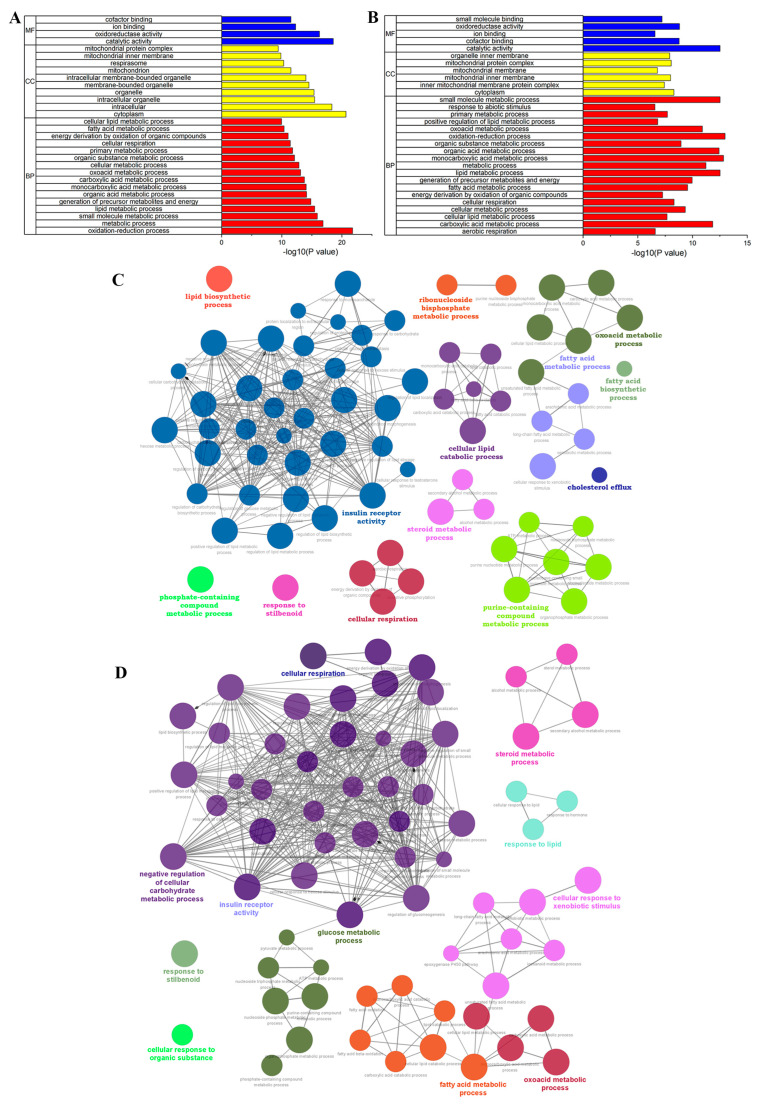
(**A**) GO analysis of DEGs in M vs. C1. (**B**) GO analysis of DEGs in M vs. C4. (**C**) ClueGO analysis of DEGs in M vs. C1. (**D**) ClueGO analysis of DEGs in M vs. C4.

Figure 4A,B demonstrate that the DEGs between the M vs. C1 and M vs. C4 groups are commonly involved in the CC classification of cytoplasm, mitochondrial inner membrane, and mitochondrial protein complex. The oxidation–reduction process, metabolic process, small-molecule metabolic process, lipid metabolic process, and generation of precursor metabolites and energy are mainly focused on in the BP classification; MF classification encompasses the catalytic activity, oxidoreductase activity, ion binding, cofactor binding, and oxidoreductase activity. The top 30 GO terms were ranked highest by the smallest *p*-value, with the BP classification in M vs. C1 and M vs. C4 accounting for 53% and 63%, respectively. Further functional analysis of DEGs in M vs. C1 and M vs. C4 for BP classification was conducted using the ClueGO plugin in Cytoscape (screening condition was to show only significant pathways, *p* < 0.05). Figure 4C,D reveal that both M vs. C1 and M vs. C4 were mainly enriched in the fatty acid metabolic process, oxoacid metabolic process, cellular respiration, insulin receptor activity, steroid metabolic process, and response to stilbenoids.

**Figure 5:**
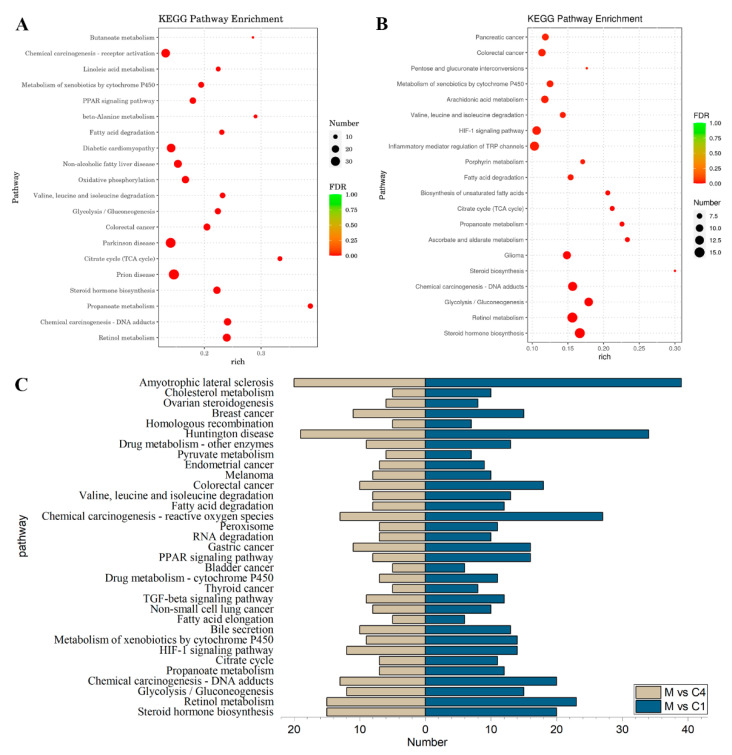
(**A**) KEGG pathway analysis of DEGs in M vs. C1. (**B**) KEGG pathway analysis of DEGs in M vs. C4. (**C**) The 33 common pathways between M vs. C1 and M vs. C4.

Furthermore, a KEGG pathway enrichment analysis was performed, with the DEGs being significantly enriched in 69 pathways for the M vs. C1 group (Supplementary Table S1) and 59 pathways for the M vs. C4 group (Supplementary Table S2). The 20 most crucial KEGG pathways were compiled and displayed according to their FDR value, sorted from low to high. Figure 5A illustrates that the DEGs were primarily enriched in retinol metabolism, chemical carcinogenesis-DNA adducts, propanoate above, steroid hormone biosynthesis, and prion disease. Meanwhile, Figure 5B reveals that the DEGs were mainly enriched in steroid hormone biosynthesis, retinol metabolism, glycolysis/gluconeogenesis, chemical carcinogenesis-DNA adducts, and steroid biosynthesis. Additionally, Figure 5C indicates that M vs. C1 and M vs. C4 shared 33 common pathways that were significantly enriched simultaneously, including the PPAR signaling pathway, steroid hormone biosynthesis, fatty acid degradation, fatty acid synthesis, and other pathways related to lipid metabolism disorders in type 2 diabetes.

### 3.7. qRT-PCR and Western Blot Validation

**Figure 6:**
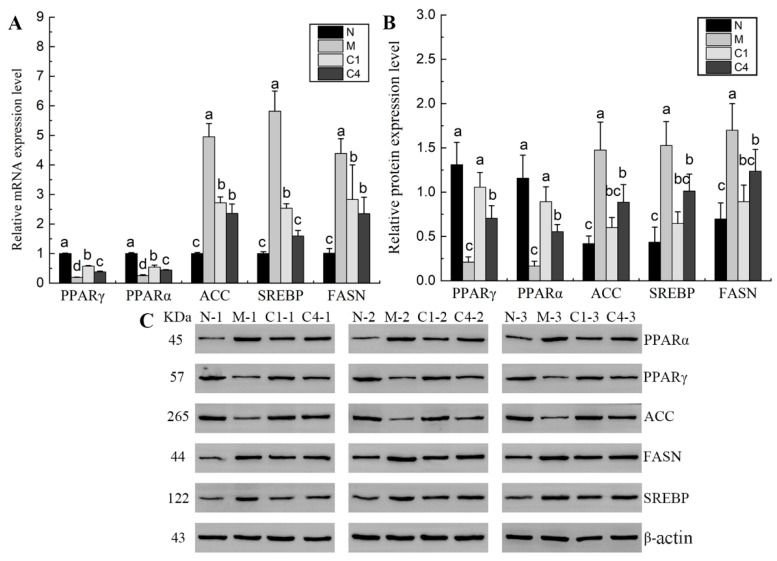
(**A**) The relative gene expression levels of PPARα, ACC, PPARγ, FASN, and SREBP. (**B**) The relative protein expression levels of PPARα, ACC, PPARγ, FASN, and SREBP. (**C**) Western blot validation of the relative protein expression levels of PPARα, PPARγ, SREBP, FASN, and ACC. All data are expressed as mean ± SD (*n* = 3). Different lowercase letters indicate significant differences (*p* < 0.05).

The gene and protein expression levels of PPARα, ACC, PPARγ, FASN, and SREBP were assessed using qRT-PCR and Western blot methods, respectively. Figure 6 demonstrates that, compared to the M group, the gene and protein expression levels of PPARα increased by 114.49% and 435.98% in the C1 group and by 75.01% and 232.61% in the C4 group. Meanwhile, compared to the M group, the gene expression levels of PPARγ in the C1 and C4 groups increased by 196.59% and 96.59%, respectively, with their protein expression levels also significantly increasing by 404.97% and 236.90%, respectively. Conversely, compared to the C1 group, the gene and protein expression levels of PPARα and PPARγ in the C4 group were significantly reduced by 18.41% and 9.72% and by 37.94% and 33.28%, respectively. Moreover, the gene and protein expression levels of SREBP, FASN, and ACC in the M group were significantly higher than those in the C1 and C4 groups. A significantly lower level of SREBP gene expression was observed in the C4 group compared to the C1 group, while their protein expression levels were significantly higher than those in the C1 group. The gene and protein expression levels of FASN and ACC in the C4 group showed no significant difference compared to the C1 group, while the protein expression levels of FASN and ACC in the C4 group were close to the level of the N Group. These findings indicate that RTT ellagitannin can significantly modulate the gene and protein expression levels of key enzymes involved in lipid synthesis, promote fatty acid oxidation, and enhance lipolysis, thereby achieving effective lipid-lowering outcomes.

## 4. Discussion

Worldwide, millions of people suffer from type 2 diabetes, and after decades of research, numerous natural active ingredients from plants have been discovered to help prevent this condition [28]. A previous study revealed that the oral administration of 50 mg/kg RTT ellagic acid and ellagitannin decreased insulin levels in db/db mice, enhanced glucose tolerance, and significantly improved liver antioxidant capacity [15]. However, the precise regulatory mechanism of lipid metabolism in db/db mice remains unclear. Therefore, our study employed transcriptomic techniques to investigate the mechanism by which RTT ellagitannin ameliorates lipid metabolism dysfunction in diabetic mice. We found that, during the 8-week intervention period, the body weight of type 2 diabetic mice increased, while RTT ellagic acid and ellagitannin intervention slowed this rate and significantly reduced FBG in these mice, with RTT ellagitannin demonstrating more substantial effects. A pathological analysis of the liver revealed that the hepatocytes of diabetic mice were severely lesioned and occupied the entire liver tissue organs. Compared with the untreated diabetic mice, the intervention experimental group significantly reduced the extent of lipid droplet deposition. In terms of alleviating lipid accumulation in the liver, metformin and RTT ellagitannin showed better improvement, while RTT ellagic acid showed poorer improvement. RTT ellagitannin intervention significantly decreased TC, TG, and LDL-C levels in the liver, although RTT ellagic acid did not notably alter LDL-C levels. Additionally, HDL-C levels in mice liver did not differ significantly between groups, but RTT ellagitannin intervention increased HDL-C levels. Consequently, RTT ellagitannin effectively alleviated lipid accumulation in db/db mice, reduced cholesterol synthesis, and managed lipid metabolism disorders.

Utilizing transcriptome sequencing technology, our analysis discovered that, in comparison to the M group, there were 1245 and 707 DEGs in the C1 and C4 groups, respectively. Approximately 537 common DEGs were expressed in both groups, which were significantly involved in the BP classification of fatty acid metabolism, oxoacid metabolism, cellular respiration, and steroid metabolic process functional annotation of the GO term and the visual functional enrichment analysis using ClueGO. Among the KEGG enrichment pathways, 33 identical pathways were found in ellagic acid and ellagitannin groups, including steroid hormone biosynthesis, PPAR signaling pathway, fatty acid degradation, and fatty acid synthesis, which are all related to lipid metabolism.

Cholesterol is crucial for steroid hormone synthesis [29]. Proteins involved in steroid hormone synthesis transport cholesterol from the outer mitochondrial membrane to the inner membrane, where it is converted to pregnenolone by rate-limiting enzymes, ultimately leading to the production of glucocorticoids [30]. Glucocorticoids play a role in regulating glucose, protein, and fat metabolism, as well as osmolarity balance. They control glycemic changes, induce insulin resistance, increase triglyceride synthesis, and decrease fatty acid oxidation by interfering with insulin signaling [31,32]. Our findings suggest that RTT ellagitannin may regulate hepatic cholesterol levels by modulating the steroid hormone biosynthesis pathway, potentially contributing to the maintenance of glucolipid homeostasis.

Fatty acid metabolism involves the uptake, synthesis, and oxidation of fatty acids. When the β-oxidation metabolic capacity is insufficient to handle circulating fatty acids, esterification occurs, resulting in the storage of fatty acids as triglycerides (TGs) in the liver and adipocytes. This process, along with long-term past interactions, leads to fat deposition [33]. The hepatic lipid metabolism is strictly regulated by SREBP and PPARs, with PPARα isoforms regulating fatty acid oxidation upon activation [34]. Elevated levels of PPARα gene expression promote mitochondrial fatty acid oxidation, which results in increased insulin secretion in response to glucose stimulation in rat β-cells [35]. PPARα activation decreases TG and LDL-C levels [36] and regulates lipid fluxes in the liver by controlling the oxidative metabolism of fatty acids and lipoproteins [37,38]. On the other hand, the PPARγ isoform primarily affects fatty acid synthesis, activates glucocorticoids, contributes to abnormal fatty acid metabolism [39], regulates triglyceride homeostasis, and mitigates hyperglycemia and hyperlipidemia damage [40]. RTT ellagitannin, in this study, increased both gene and protein expression levels of PPARα and PPARγ, acting as dual agonists. The simultaneous activation of these two isoforms better combined lipid metabolism and insulin secretion, resulting in hypoglycemic and hypolipidemic effects. These results were consistent with Han et al. [41]. Furthermore, PPARs activation facilitated hepatic cholesterol efflux and reduced glucose levels in the liver of db/db mice [41,42].

SREBP is primarily involved in fatty acid uptake and *de novo* synthesis in lipid metabolism. It promotes lipid deposition by enhancing the expression of key genes (*FASN* and *ACC*) that regulate downstream lipid synthesis, contributing to triglyceride and lipid droplet formation [43,44,45,46]. FASN catalyzes the conversion of acetyl coenzyme A into intermediate products (saturated fatty acids), which are then esterified to produce fats [47]. Mehmood et al. [48] found that ACNs stimulated lipid oxidation in mitochondria by inhibiting ACC rate-limiting enzyme activity through phosphorylation and controlling malonyl coenzyme A synthesis. In the model group, the *SREBP* gene was upregulated, and downstream genes of lipid synthesis FASN and ACC were overexpressed. Elevated concentrations of TG activated downstream forms that competed with glucose to enter the cell, resulting in lipotoxic insulin resistance. Excess free fatty acids contributed to ectopic lipid deposition, increased glycolysis, and inhibition of glycogen synthesis, ultimately leading to decreased insulin sensitivity [49,50]. The present study is consistent with Zhang et al. [51], demonstrating that ellagitannin significantly reduced *SREBP* expression in the liver, inhibited *FASN* and *ACC* gene and protein expression, and slowed down fatty acid and triglyceride synthesis. This led to the prevention of hepatic fat accumulation and liver protection. The protein expression of SREBP in the ellagic acid and ellagitannin groups was inconsistent with the trend of gene expression. The peak of protein expression may be reached when the gene is degraded, and the difference in trend could be due to transcriptional and translational space–time sites [52,53]. Compared to ellagic acid, RTT ellagitannin reduced hepatic lipid deposition with better inhibition of lipid metabolism disorders in db/db mice. Thus, RTT ellagitannin holds better potential for regulating lipid metabolism homeostasis in type 2 diabetes.

## 5. Conclusions

In conclusion, RTT ellagitannin exhibited a greater capacity than ellagic acid to improve FBG and hepatic lipid levels in type 2 db/db mice. Moreover, it was significantly enriched in the KEGG enrichment pathways of steroid hormone biosynthesis, PPAR signaling, fatty acid degradation, and fatty acid synthesis. RTT ellagitannin significantly increased the gene and protein expression levels of PPARα and PPARγ, while downregulating the gene and protein expression levels of SREBP, FASN, and ACC. These findings suggest that RTT ellagitannin could serve as an active ingredient for improving lipid metabolism disorders in type 2 diabetes.

## Figures and Tables

**Table 1:** Genetic primer sequence information.

Gene Names	Forward Primer (5′-3′)	Reverse Primer (5′-3′)
*PPARα*	AATGAACGTGCAATCCGATTTG	ACTCCACATTTGCGTAATTGTTG
*PPARγ*	GGAAGACCACTCGCATTCCTT	GTAATCAGCAACCATTGGGTCA
*ACC*	AATGAACGTGCAATCCGATTTG	ACTCCACATTTGCGTAATTGTTG
*FASN*	AGGTGGTGATAGCCGGTATGT	TGGGTAATCCATAGAGCCCAG
*SREBP*	CTTTGGCCTCGCTTTTCGG	TGGGTCCAATTAGAGCCATCTC
*β-Actin*	GTGCTATGTTGCTCTAGACTTCG	ATGCCACAGGATTCCATACC

**Table 2:** Effect of ellagitannin on body weight in mice (*n* = 6).

Time (Week)	Body Weight (g) *				
	N	M	P	C1	C4
1	20.18 ± 1.08 ^b^	33.11 ± 0.13 ^a^	33.81 ± 1.01 ^a^	33.41 ± 0.56 ^a^	33.27 ± 0.96 ^a^
2	21.76 ± 0.54 ^c^	42.09 ± 0.92 ^a^	40.67 ± 0.95 ^ab^	39.53 ± 1.05 ^b^	39.36 ± 1.10 ^b^
3	21.56 ± 2.00 ^c^	45.10 ± 0.60 ^a^	41.28 ± 1.44 ^b^	41.88 ± 0.97 ^b^	42.65 ± 0.94 ^ab^
4	23.31 ± 1.42 ^c^	47.61 ± 0.70 ^a^	43.43 ± 0.81 ^b^	43.81 ± 1.01 ^b^	45.24 ± 0.70 ^b^
5	23.30 ± 1.18 ^c^	49.86 ± 1.46 ^a^	46.42 ± 1.14 ^b^	46.39 ± 0.69 ^b^	48.45 ± 0.93 ^ab^
6	24.37 ± 1.06 ^c^	53.31 ± 1.44 ^a^	49.23 ± 0.83 ^c^	48.25 ± 0.96 ^c^	52.58 ± 1.30 ^b^
7	24.54 ± 0.89 ^e^	55.56 ± 0.90 ^a^	51.52 ± 1.01 ^c^	49.30 ± 0.95 ^d^	53.38 ± 0.89 ^b^
8	24.43 ± 0.71 ^c^	55.51 ± 0.62 ^a^	51.44 ± 1.34 ^b^	50.64 ± 1.14 ^b^	52.63 ± 1.23 ^b^

* Different lowercase letters indicate significant differences between treatments of the same week (*p* < 0.05).

**Table 3:** Effect of ellagitannin on FBG in mice (*n* = 6).

Time (Week)	FBG (mmol/L) *				
	N	M	P	C1	C4
0	4.58 ± 1.16 ^b^	9.84 ± 2.67 ^a^	9.05 ± 1.43 ^a^	9.45 ± 1.09 ^a^	9.64 ± 0.91 ^a^
4	5.37 ± 0.66 ^c^	20.14 ± 4.92 ^ab^	23.13 ± 7.40 ^a^	12.87 ± 0.31 ^bc^	14.57 ± 3.13 ^b^
8	5.38 ± 0.92 ^c^	21.77 ± 4.06 ^a^	14.93 ± 3.84 ^b^	10.39 ± 0.73 ^bc^	11.73 ± 1.91 ^b^

* Different lowercase letters indicate significant differences between treatments of the same week (*p* < 0.05).

**Table 4:** RNS-seq quality control base data statistics.

Sample	Reads No.	Bases (bp)	Q30 (bp)	N (%)	Q20 (%) *	Q30 (%) *
M_1	49989464	7498419600	6995602942	0.000356	97.36	93.29
M_2	54246640	8136996000	7628038842	0.000342	97.58	93.74
M_3	45603244	6840486600	6382261682	0.000341	97.37	93.3
C1_1	52733304	7909995600	7408232317	0.000355	97.55	93.65
C1_2	51074184	7661127600	7183401187	0.00036	97.6	93.76
C1_3	43787058	6568058700	5993737424	0.000129	96.73	91.25
C4_1	44626308	6693946200	6266481249	0.000357	97.47	93.61
C4_2	45570930	6835639500	6405171513	0.000356	97.54	93.7
C4_3	56577088	8486563200	7934581869	0.000347	97.47	93.49

* Q20 represents the percentage of bases with base recognition accuracy of 99% or more; Q30 represents the percentage of bases with base identification accuracy of 99.9% or more.

## Data Availability

Data for the results of this study are available from the corresponding author upon reasonable request.

## Supplementary Materials

The following supporting information can be downloaded at https://www.mdpi.com/article/10.3390/nu15194187/s1, Table S1: KEGG enrichment pathway of DEGs in M vs. C1; Table S2: KEGG enrichment pathway of DEGs in M vs. C4.
