# Supplementary material for: Transcriptomics Reveals the Mechanism of Rosa roxburghii Tratt Ellagitannin in Improving Hepatic Lipid Metabolism Disorder in db/db Mice
Source: Nutrients. 2023 Sep 28;15(19):4187. doi: 10.3390/nu15194187 (PMC10574348; doi:10.3390/nu15194187)
Supplement: Supplementary file 1 [file nutrients-15-04187-s001.zip › nutrients-2589780-supplementary.pdf]

Table S1. KEGG enrichment pathway of DEGs in M vs C1.

PathwayID	Pathway	Level1	Level2	DEG_number	total_number	Pvalue	FDR
mmu00830	Retinol metabolism	Metabolism	Metabolism of cofactors and vitamins	23	96	1.98165E-08	6.32145E-06
mmu05204	Chemical carcinogenesis - DNA adducts	Human Diseases	Cancer: overview	20	83	1.51619E-07	1.87647E-05
mmu00640	Propanoate metabolism	Metabolism	Carbohydrate metabolism	12	31	1.76471E-07	1.87647E-05
mmu00140	Steroid hormone biosynthesis	Metabolism	Lipid metabolism	20	90	6.2126E-07	4.95455E-05
mmu05020	Prion disease	Human Diseases	Neurodegenerative disease	37	253	1.37308E-06	8.76026E-05
mmu00020	Citrate cycle (TCA cycle)	Metabolism	Carbohydrate metabolism	11	33	3.28274E-06	0.000174532
mmu05012	Parkinson disease	Human Diseases	Neurodegenerative disease	35	249	6.6217E-06	0.00030176
mmu05210	Colorectal cancer	Human Diseases	Cancer: specific types	18	88	7.90276E-06	0.000315122
mmu00010	Glycolysis / Gluconeogenesis	Metabolism	Carbohydrate metabolism	15	67	1.43836E-05	0.000509818
mmu00280	Valine, leucine and isoleucine degradation	Metabolism	Amino acid metabolism	13	56	3.55507E-05	0.001134067
mmu00190	Oxidative phosphorylatio	Metabolism	Energy metabolism	20	120	5.97289E-05	0.001672601
mmu04932	Non-alcoholic fatty liver disease	Human Diseases	Endocrine and metabolic disease	23	150	6.62751E-05	0.001672601

mmu05415	Diabetic cardiomyopath	Human Diseases	Cardiovascular disease	27	191	6.81624E-05	0.001672601
mmu00071	Fatty acid degradation	Metabolism	Lipid metabolism	12	52	7.54144E-05	0.001718372
mmu00410	beta-Alanine metabolism β -	Metabolism	Metabolism of other amino acids	9	31	8.96213E-05	0.001905945
mmu03320	PPAR signaling pathway	Organismal Systems	Endocrine system	16	89	0.000129573	0.002583353
mmu00980	Metabolism of xenobiotics by cytochrome P450	Metabolism	Xenobiotics biodegradation and metabolism	14	72	0.000142615	0.00267612
mmu00591	Linoleic acid metabolism亚	Metabolism	Lipid metabolism	11	49	0.000194965	0.003455211
mmu05207	Chemical carcinogenesis - receptor activation	Human Diseases	Cancer: overview	27	205	0.000227966	0.003827429
mmu00650	Butanoate metabolism	Metabolism	Carbohydrate metabolism	8	28	0.00025093	0.004002328
mmu05208	Chemical carcinogenesis - reactive oxygen species	Human Diseases	Cancer: overview	27	207	0.000267587	0.004064778
mmu05016	Huntington disease	Human Diseases	Neurodegenerative disease	34	287	0.000296077	0.00429312
mmu05014	Amyotrophic lateral sclerosis	Human Diseases	Neurodegenerative disease	39	351	0.000409662	0.005610509
mmu00053	Ascorbate and aldarate metabolism	Metabolism	Carbohydrate metabolism	8	30	0.000422107	0.005610509
mmu00590	Arachidonic acid metabolism	Metabolism	Lipid metabolism	14	85	0.000846212	0.010402085
mmu04979	Cholesterol	Organismal	Digestive system	10	49	0.000847819	0.010402085

	metabolism	Systems					
mmu05212	Pancreatic cancer	Human Diseases	Cancer: specific types	13	76	0.000892067	0.010539612
mmu05010	Alzheimer disease	Human Diseases	Neurodegenerative disease	39	367	0.000981132	0.011177893
mmu01040	Biosynthesis of unsaturated fatty acids	Metabolism	Lipid metabolism	8	34	0.001045466	0.011500127
mmu04714	Thermogenesis	Organismal Systems	Environmental adaptation	26	215	0.001102054	0.011718504
mmu03420	Nucleotide excision repair	Genetic Information Processing	Replication and repair	9	43	0.0012656	0.013023434
mmu05216	Thyroid cancer	Human Diseases	Cancer: specific types	8	37	0.001878724	0.018728532
mmu00860	Porphyrin metabolism	Metabolism	Metabolism of cofactors and vitamins	8	41	0.003717525	0.035936071
mmu00982	Drug metabolism - cytochrome P450	Metabolism	Xenobiotics biodegradation and metabolism	11	70	0.00432029	0.040534484
mmu00983	Drug metabolism - other enzymes	Metabolism	Xenobiotics biodegradation and metabolism	13	91	0.004688224	0.042459231
mmu00040	Pentose and glucuronate interconversions	Metabolism	Carbohydrate metabolism	7	34	0.004791637	0.042459231
mmu05214	Glioma	Human Diseases	Cancer: specific types	11	74	0.006639461	0.057242917
mmu05205	Proteoglycans in cancer	Human Diseases	Cancer: overview	22	201	0.008540734	0.070664139
mmu00062	Fatty acid elongation	Metabolism	Lipid metabolism	6	29	0.008639189	0.070664139
mmu05022	Pathways of neurodegeneration -	Human Diseases	Neurodegenerative disease	42	456	0.008993488	0.071723064

	multiple diseases						
mmu04976	Bile secretion	Organismal Systems	Digestive system	13	99	0.009531903	0.074162852
mmu05213	Endometrial cancer	Human Diseases	Cancer: specific types	9	58	0.010259488	0.077923254
mmu03430	Mismatch repair	Genetic Information Processing	Replication and repair	5	22	0.010820909	0.080276042
mmu00260	Glycine, serine and threonine metabolism	Metabolism	Amino acid metabolism	7	40	0.011952932	0.08340345
mmu04066	HIF-1 signaling pathway	Environmental Information Processing	Signal transduction	14	113	0.011987483	0.08340345
mmu00630	Glyoxylate and dicarboxylate metabolism	Metabolism	Carbohydrate metabolism	6	31	0.01202683	0.08340345
mmu00604	Glycosphingolipid biosynthesis - ganglio series	Metabolism	Glycan biosynthesis and metabolism	4	15	0.012510605	0.084912407
mmu04750	Inflammatory mediator regulation of TRP channels	Organismal Systems	Sensory system	15	126	0.013466184	0.088854244
mmu03440	Homologous recombination	Genetic Information Processing	Replication and repair	7	41	0.013648458	0.088854244
mmu00380	Tryptophan metabolism	Metabolism	Amino acid metabolism	8	51	0.014216114	0.090698807
mmu05218	Melanoma	Human Diseases	Cancer: specific types	10	72	0.014972075	0.091847922
mmu05223	Non-small cell lung cancer	Human Diseases	Cancer: specific types	10	72	0.014972075	0.091847922
mmu04260	Cardiac muscle	Organismal	Circulatory system	11	83	0.015361752	0.092318535

	contraction	Systems					
mmu00310	Lysine degradation	Metabolism	Amino acid metabolism	9	62	0.015627589	0.092318535
mmu04350	TGF-beta signaling pathway	Environmental Information Processing	Signal transduction	12	95	0.016668339	0.096676365
mmu04146	Peroxisome	Cellular Processes	Transport and catabolism	11	86	0.01964142	0.111885945
mmu00620	Pyruvate metabolism	Metabolism	Carbohydrate metabolism	7	45	0.022163112	0.124035659
mmu05226	Gastric cancer	Human Diseases	Cancer: specific types	16	148	0.025313362	0.139223492
mmu00920	Sulfur metabolism	Metabolism	Energy metabolism	3	11	0.028639539	0.154847679
mmu04270	Vascular smooth muscle contraction	Organismal Systems	Circulatory system	15	141	0.033673804	0.174940229
mmu03018	RNA degradation	Genetic Information Processing	Folding, sorting and degradation	10	82	0.034086174	0.174940229
mmu00770	Pantothenate and CoA biosynthesis	Metabolism	Metabolism of cofactors and vitamins	4	20	0.034549324	0.174940229
mmu00100	Steroid biosynthesis	Metabolism	Lipid metabolism	4	20	0.034549324	0.174940229
mmu02010	ABC transporters	Environmental Information Processing	Membrane transport	7	50	0.037228499	0.1855608
mmu05219	Bladder cancer	Human Diseases	Cancer: specific types	6	41	0.043141105	0.211723271
mmu04913	Ovarian steroidogenesis	Organismal Systems	Endocrine system	8	63	0.044750753	0.216295304
mmu05224	Breast cancer	Human Diseases	Cancer: specific types	15	147	0.046053964	0.219271858

mmu05225	Hepatocellular carcinoma	Human Diseases	Cancer: specific types	17	173	0.047746809	0.223988707

Table S2. KEGG enrichment pathway of DEGs in M vs C4.

PathwayID	Pathway	Level1	Level2	DEG_number	total_number	Pvalue	FDR
mmu00140	Steroid hormone biosynthesis	Metabolism	Lipid metabolism	15	90	1.04824E-06	0.000314472
mmu00830	Retinol metabolism	Metabolism	Metabolism of cofactors and vitamins	15	96	2.44171E-06	0.000366257
mmu00010	Glycolysis / Gluconeogenesis	Metabolism	Carbohydrate metabolism	12	67	5.89068E-06	0.000589068
mmu05204	Chemical carcinogenesis - DNA adducts	Human Diseases	Cancer: overview	13	83	1.12277E-05	0.000842076
mmu00100	Steroid biosynthesis	Metabolism	Lipid metabolism	6	20	6.57898E-05	0.003947388
mmu05214	Glioma	Human Diseases	Cancer: specific types	11	74	8.74776E-05	0.004039549
mmu00053	Ascorbate and aldarate metabolism	Metabolism	Carbohydrate metabolism	7	30	9.42561E-05	0.004039549
mmu00640	Propanoate metabolism	Metabolism	Carbohydrate metabolism	7	31	0.000117879	0.004420464
mmu00020	Citrate cycle (TCA cycle)	Metabolism	Carbohydrate metabolism	7	33	0.00017954	0.005984674
mmu01040	Biosynthesis of unsaturated fatty acids	Metabolism	Lipid metabolism	7	34	0.000218915	0.006567446
mmu00071	Fatty acid degradation	Metabolism	Lipid metabolism	8	52	0.000637162	0.017377137
mmu00860	Porphyrin metabolism	Metabolism	Metabolism of cofactors and vitamins	7	41	0.000730393	0.018259829
mmu04750	Inflammatory mediator regulation of TRP channels	Organismal Systems	Sensory system	13	126	0.000851929	0.019659889
mmu04066	HIF-1 signaling pathway	Environmental Information Processing	Signal transduction	12	113	0.001037283	0.021122627
mmu00280	Valine, leucine and isoleucine degradation	Metabolism	Amino acid metabolism	8	56	0.001056131	0.021122627

mmu00590	Arachidonic acid metabolism	Metabolism	Lipid metabolism	10	85	0.001228945	0.023042714
mmu00980	Metabolism of xenobiotics by cytochrome P450	Metabolism	Xenobiotics biodegradation and metabolism	9	72	0.001389282	0.024428046
mmu00040	Pentose and glucuronate interconversions	Metabolism	Carbohydrate metabolism	6	34	0.001465683	0.024428046
mmu05210	Colorectal cancer	Human Diseases	Cancer: specific types	10	88	0.001605677	0.025352799
mmu05212	Pancreatic cancer	Human Diseases	Cancer: specific types	9	76	0.002040245	0.030603682
mmu00591	Linoleic acid metabolism	Metabolism	Lipid metabolism	7	49	0.002159812	0.030854458
mmu04976	Bile secretion	Organismal Systems	Digestive system	10	99	0.003859012	0.052622889
mmu00062	Fatty acid elongation	Metabolism	Lipid metabolism	5	29	0.004061727	0.052979044
mmu05207	Chemical carcinogenesis - receptor activation	Human Diseases	Cancer: overview	16	205	0.004354403	0.054430036
mmu05218	Melanoma	Human Diseases	Cancer: specific types	8	72	0.005304519	0.059458257
mmu05223	Non-small cell lung cancer	Human Diseases	Cancer: specific types	8	72	0.005304519	0.059458257
mmu04922	Glucagon signaling pathway	Organismal Systems	Endocrine system	10	104	0.005487017	0.059458257
mmu05213	Endometrial cancer	Human Diseases	Cancer: specific types	7	58	0.005674589	0.059458257
mmu05012	Parkinson disease	Human Diseases	Neurodegenerative disease	18	249	0.005747631	0.059458257
mmu00620	Pyruvate metabolism	Metabolism	Carbohydrate metabolism	6	45	0.006286586	0.062865865
mmu00983	Drug metabolism - other enzymes	Metabolism	Xenobiotics biodegradation and metabolism	9	91	0.006885007	0.0666291
mmu04350	TGF-beta signaling pathway	Environmental Information Processing	Signal transduction	9	95	0.009065432	0.084988421
mmu05016	Huntington disease	Human Diseases	Neurodegenerative disease	19	287	0.011444892	0.103085242
mmu05216	Thyroid cancer	Human Diseases	Cancer: specific types	5	37	0.011682994	0.103085242
mmu05415	Diabetic cardiomyopathy	Human Diseases	Cardiovascular disease	14	191	0.012539673	0.107482909

mmu05020	Prion disease	Human Diseases	Neurodegenerative disease	17	253	0.014239285	0.114966508
mmu00190	Oxidative phosphorylation	Metabolism	Energy metabolism	10	120	0.014465174	0.114966508
mmu04015	Rap1 signaling pathway	Environmental Information Processing	Signal transduction	15	214	0.014562424	0.114966508
mmu00982	Drug metabolism - cytochrome P450	Metabolism	Xenobiotics biodegradation and metabolism	7	70	0.015473464	0.119026645
mmu03440	Homologous recombination	Genetic Information Processing	Replication and repair	5	41	0.01779389	0.129644408
mmu05219	Bladder cancer	Human Diseases	Cancer: specific types	5	41	0.01779389	0.129644408
mmu03320	PPAR signaling pathway	Organismal Systems	Endocrine system	8	89	0.018150217	0.129644408
mmu05224	Breast cancer	Human Diseases	Cancer: specific types	11	147	0.022114417	0.154146575
mmu04310	Wnt signaling pathway	Environmental Information Processing	Signal transduction	12	167	0.022975065	0.154146575
mmu05226	Gastric cancer	Human Diseases	Cancer: specific types	11	148	0.023121986	0.154146575
mmu04932	Non-alcoholic fatty liver disease	Human Diseases	Endocrine and metabolic disease	11	150	0.025237293	0.164591039
mmu00410	beta-Alanine metabolism	Metabolism	Metabolism of other amino acids	4	31	0.027468771	0.171811837
mmu04921	Oxytocin signaling pathway	Organismal Systems	Endocrine system	11	152	0.027489894	0.171811837
mmu01521	EGFR tyrosine kinase inhibitor resistance	Human Diseases	Drug resistance: antineoplastic	7	79	0.028198189	0.172641971
mmu04913	Ovarian steroidogenesis	Organismal Systems	Endocrine system	6	63	0.030017806	0.180106835
mmu05215	Prostate cancer	Human Diseases	Cancer: specific types	8	99	0.032011998	0.188305872
mmu03018	RNA degradation	Genetic Information Processing	Folding, sorting and degradation	7	82	0.033668898	0.194243644
mmu04979	Cholesterol metabolism	Organismal Systems	Digestive system	5	49	0.035592234	0.201465478

mmu05014	Amyotrophic lateral sclerosis	Human Diseases	Neurodegenerative disease	20	351	0.040549392	0.225274402
mmu04146	Peroxisome	Cellular Processes	Transport and catabolism	7	86	0.042002018	0.22846068
mmu04152	AMPK signaling pathway	Environmental Information Processing	Signal transduction	9	124	0.043055115	0.22846068
mmu05163	Human cytomegalovirus infection	Human Diseases	Infectious disease: viral	15	246	0.043407529	0.22846068
mmu05208	Chemical carcinogenesis - reactive oxygen species	Human Diseases	Cancer: overview	13	207	0.047429894	0.245327036
mmu04010	MAPK signaling pathway	Environmental Information Processing	Signal transduction	17	293	0.048855479	0.248417692
